# Retraction: Sinomenine Sensitizes Multidrug-Resistant Colon Cancer Cells (Caco-2) to Doxorubicin by Downregulation of MDR-1 Expression

**DOI:** 10.1371/journal.pone.0215388

**Published:** 2019-04-09

**Authors:** Zhen Liu, Zhi-Jun Duan, Jiu-Yang Chang, Zhi-feng Zhang, Rui Chu, Yu-Ling Li, Ke-Hang Dai, Guang-quan Mo, Qing-Yong Chang, and the PLOS ONE Editors

Following publication of this article [1], the following concerns were raised:

1. The Beta-Actin panel, lanes 2–4, in Fig 4B is similar to the Beta-Actin panel, lanes 1–3, in Fig 6B. The lanes represent different experimental conditions in the two figures.
2. The Beta-Actin panel in Fig 5A is similar to the IκB-α panel in Fig 6B.

These concerns also call into question the validity of the quantification data shown in Figs 4B, 5B, and 6C.

The authors were approached for comments shortly after publication and they noted that the underlying data supporting these figures are no longer available. The authors requested retraction of the article at the time. The *PLOS ONE* Editors apologize for the journal’s delay in completing our follow-up after receiving this request.

The authors and *PLOS ONE* Editors retract this article in light of the concerns raised and the unavailability of underlying data to support the results reported in the article.
